# Impact of Nutrient Stress on Plant Disease Resistance

**DOI:** 10.3390/ijms26041780

**Published:** 2025-02-19

**Authors:** Héctor Martín-Cardoso, Blanca San Segundo

**Affiliations:** 1 Centre for Research in Agricultural Genomics (CRAG) CSIC-IRTA-UAB-UB, Campus Universitat Autònoma de Barcelona, Bellaterra (Cerdanyola del Vallès), 08193 Barcelona, Spain; hector.martin@cragenomica.es; 2 Consejo Superior de Investigaciones Científicas (CSIC), 08193 Barcelona, Spain

**Keywords:** abiotic stress, biotic stress, disease resistance, microRNAs, nutrients, pathogens, plant immunity

## Abstract

Plants are constantly exposed to abiotic and biotic stresses that seriously affect crop yield and quality. A coordinated regulation of plant responses to combined abiotic/biotic stresses requires crosstalk between signaling pathways initiated by each stressor. Interconnected signaling pathways further finetune plant stress responses and allow the plant to respond to such stresses effectively. The plant nutritional status might influence disease resistance by strengthening or weakening plant immune responses, as well as through modulation of the pathogenicity program in the pathogen. Here, we discuss advances in our understanding of interactions between nutrient stress, deficiency or excess, and immune signaling pathways in the context of current agricultural practices. The introduction of chemical fertilizers and pesticides was a major component of the Green Revolution initiated in the 1960s that greatly boosted crop production. However, the massive application of agrochemicals also has adverse consequences on the environment and animal/human health. Therefore, an in-depth understanding of the connections between stress caused by overfertilization (or low bioavailability of nutrients) and immune responses is a timely and novel field of research with important implications for disease control in crop species. Optimizing nutrient management practices tailored to specific environmental conditions will be crucial in maximizing crop production using environmentally friendly systems.

## 1. Introduction

In nature, plants are constantly exposed to a multitude of biotic and abiotic stresses that can limit plant growth and productivity. Biotic stress can be caused by fungi, bacteria, viruses, insect pests, nematodes, or parasitic plants, while abiotic stress comprises drought, salinity, high/low temperatures, flooding, heavy metals, or nutritional stress, among others. Any of these stresses might have detrimental effects on plant growth and productivity by interfering with normal physiological, biochemical, and molecular functions. To cope with environmental stresses, plants have evolved multiple strategies that can take place at the cellular, organ, and whole-plant levels. Multiple studies have so far been carried out to investigate plant adaptive responses to environmental stressors focused on plants exposed to an individual stress [1,2,3]. Under field conditions, however, plants are often challenged by combinations of abiotic and biotic stresses, and the plant response to stress combinations differs from that triggered by each stress individually [4].

Upon perception of a particular stimuli, multiple signaling cascades are activated, leading to transcriptional reprogramming, a response that allows the plant to generate appropriate responses. It is increasingly apparent that signaling pathways activated by a stressful factor are not linear pathways but are part of a more complex network in which signaling pathways are induced by different types of stress converge. The effect of combined stress factors is dependent on the nature of interactions between stress factors, as different types of stress interactions can have diverse effects on plants. For instance, abiotic stress might influence plant–pathogen interactions either positively or negatively, thereby enhancing or decreasing the severity of disease [5,6,7]. At present, there is still a lack of knowledge regarding plant responses to combined abiotic and biotic stress in plants.

Nutrient stress refers to either deficiency or excess of essential nutrients. A proper balance of nutrients is important not only for maintaining plant fitness but might also influence disease resistance [8,9]. A priori, healthy plants which certainly have high vigor would be expected to have improved tolerance to environmental stress compared to unhealthy plants, while nutrient deficiency might negatively impact stress tolerance [10]. However, variable effects can be found in the literature on enhancing or decreasing resistance or susceptibility to pathogen infection depending on the nutrient and the host–pathogen interaction [11,12,13]. Increasing evidence also indicates that nutrient availability might affect plant disease resistance in various ways, acting not only on the plant side, but also on the pathogen side [12,13,14,15,16,17,18,19,20,21,22,23].

In this review, we present an overview of the impact of nutritional stress in plant disease resistance. Food security faces a number of challenges, including climate change, water scarcity, soil degradation, and diseases. One key question refers to the way by which abiotic stresses might affect disease resistance in plants, and how the plant nutritional status might condition of host immune responses and/or pathogen virulence. To facilitate the analysis, we first summarized mechanisms regulating plant immunity against pathogen infection (e.g., bacterial and fungal pathogens). The impact of plant nutrition on disease resistance is presented mainly, but not exclusively, in rice and Arabidopsis plants, which are the model systems for research on monocotyledonous and dicotyledonous plant species, respectively. The sequencing of the Arabidopsis and rice genomes 20 years ago opened up new avenues for investigations into molecular genetics, with these species subsequently dominating plant research. *Arabidopsis thaliana* is not an economically important plant, but it has been the focus of intense genetic, physiological, biochemical, and molecular studies. Arabidopsis is closely related to economically important crop plants, such as cabbage, broccoli, turnip, and canola. Due to its scientific and practical advantages (e.g., short generation time, relatively small genome size, well curated and annotated genome sequence, availability of mutant and gene collections, amenability to genetic modifications, etc.), Arabidopsis is universally acknowledged as the model for dicotyledonous crop plants. However, even though the study of Arabidopsis has enabled us to gain a fundamental understanding of plant biology, not all this knowledge can be directly transferrable to monocots, such as processes which are relevant to crop production [24]. Monocots, which include economically and culturally important crops, such as cereals, account for most of the staple foods of the world. In this context, significant advances have been experienced in the past years in our understanding on the rice biology and development of resources as well as in technological advances for genetic improvement. Rice is now recognized as the model species for functional genomics and biotechnological applications in monocotyledonous species, including cereals. This review also accentuates the importance of microRNAs (miRNAs) in modulating plant responses to pathogen infection under nutrient stress conditions. The impact of cross-kingdom regulation of gene expression by small RNAs, including miRNAs, on disease resistance is also presented. Lastly, we discuss the impact of inadequate use of fertilizers and pesticides on plant disease in modern agriculture.

## 2. Abiotic and Biotic Stresses in Plants

### 2.1. Abiotic Stress in Plants

**Figure 1:**
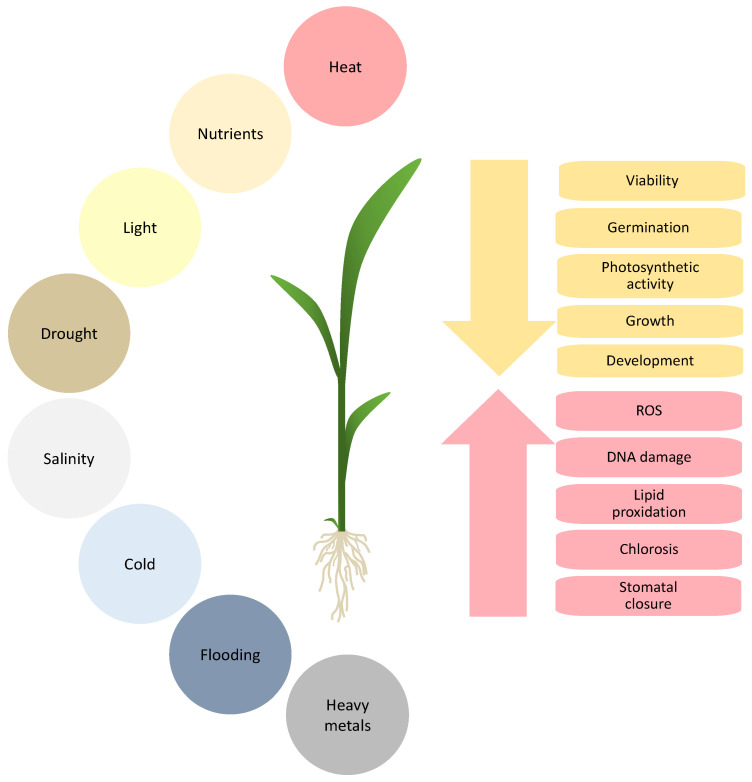
Major abiotic stresses in plants and their effects on physiological and biochemical processes affecting plant growth and development.

In nature, plants are constantly subjected to a wide range of abiotic factors, such as drought, salinity, extreme temperatures (heat, cold), nutritional deficiency, flooding, light stress (UV radiation), and chemical pollutants [25]. Major abiotic stress-related factors and the effects they can have on physiological and biochemical processes in plants are illustrated in Figure 1. Furthermore, the combination of abiotic stresses might have overwhelming impacts on the growth and productivity of crops [4]. Two abiotic stresses occurring simultaneously can have either additive negative or positive effects on plant growth and survival compared to the observed responses when those stresses are applied individually. For instance, the simultaneous application of drought and ozone to a *Medicago truncatula* cultivar that is sensitive to ozone and drought stress, when applied in combination, increased tolerance to these stressors [26]. As an example of positive interaction between abiotic stresses, elevated CO_2_ and its combination with salinity or high light increased biomass production in lettuce [27]. Furthermore, global climate change is predicted to increase the frequency and intensity of these abiotic stresses, which could lead to important losses in agriculture. This situation will pose new challenges in ensuring food security worldwide.

Plant responses to abiotic stress have been the subject of numerous studies, initially on model plants (e.g., *Arabidopsis thaliana*) and, more recently, on economically important crops (e.g., rice, corn, and soybean, among others). Generally speaking, upon perception of abiotic stress, plants redirect energy and nutrients to reproduction and defense mechanisms, which results in a decrease in biomass production [28]. Shared and unique responses occur in plants to abiotic stresses. Although very different, a number of mechanisms are common in the plant response to drought, salinity, heat and cold stress, including alterations in intracellular Ca^2+^, the production of reactive oxygen species (ROS) and oxidative stress, membrane damage, protein denaturation, and the initiation of protein phosphorylation cascades [29,30,31].

#### 2.1.1. Abiotic Environmental Stresses: Drought, Salt, Cold, and Heat Stress

Drought stress, caused by lack of precipitation or irrigation, is one of the major abiotic stresses in agricultural systems. Drought imposes a water deficit, thus reducing growth and yield in crops. Plants respond to a water deficit with stomatal closure and acceleration of photoreduction of oxygen in the chloroplast eventually leading to oxidative damage due to the accumulation of ROS [32]. Under drought conditions, plants also produce and accumulate increased amounts of abscisic acid (ABA) in the guard cells, and this induces stomatal closure to conserve water. As ABA also plays a crucial role in plant responses to pathogen infection, drought-induced ABA signaling might impact disease resistance in plants [33]. Drought stress might also result from other environmental factors, such as heat, which would then exacerbate its impact on plants. Moreover, drought might lead to reduced plant uptake of nutrients from the soil leading to nutrient stress in the plant [34]. Certainly, climate change also contributes to drought as warmer temperatures enhance evaporation and dries out soil and vegetation. As rice is mainly cultivated under flooded conditions (paddy fields), water scarcity due to climate change is predicted to have a great impact on rice production during the next years [35].

Salt stress is considered a major abiotic stress affecting crop productivity worldwide. Salinity imposes two types of stress in plants, namely osmotic and ionic stress. Thus, it causes an increase in intracellular osmotic pressure and ion toxicity due to intracellular Na^+^ accumulation [36,37]. A high salt concentration in the soil reduces water uptake by plant roots, generating water deficit or drought stress [38]. Along with this, common physiological, biochemical, and molecular responses occur in plants during salt and drought stress, in which signaling pathways induced by one or another stress are interconnected at many levels [30]. Molecular mechanisms governing salt and drought stress responses in plants have been recently summarized [39,40]. The accumulation of ions as Na^+^ and Cl^−^ might also provoke nutritional disorders in the plant. Salinity also causes hormonal imbalances and oxidative stress, that might affect susceptibility to diseases. On the other hand, soil salinization is progressively increasing in arable land for several reasons at the global level, including low precipitation, high surface evaporation, industrial pollution, and poor irrigation practices or inappropriate agrochemical usage. Among cereals, rice is highly sensitive to salinity, with higher sensitivity during early seedling and reproductive stages. Salt stress reduces growth, tiller number, panicle number, spikelets per panicle, and grain-filling in rice, thus resulting in reduced grain yield [41].

It is also true that the application of mild salinity of moderate drought stress might have a positive impact on plant growth. Mild to moderate drought stress was reported to stimulate synthetic microbial communities (SynComs) to promote growth of the forage species *Neopallasia pectinata* [42]. From the available evidence, it also appears that short-term irrigation with moderately saline water reduces the impact of drought stress in important food security crops, such as taro, yam and pumpkin [43].

Extreme temperatures influence many physiological functions in plants. There are certain developmental stages that exhibit heightened sensitivity to elevated temperatures (e.g., early establishment, flowering, and gametogenesis) [44]. Mechanisms governing heat and cold stress responses in plants have been recently reviewed [40,45]. However, an increase in temperature is not always detrimental to plants, as mild to moderate temperature elevations can induce a developmental transition referred to as thermo-morphogenesis. This phenomenon is characterized by the elongation of the hypocotyl, alterations in flowering, and morphological and architectural transformations [46]. Plants can also adapt to low-temperature environments, a process termed cold acclimation, in which exposition to temperatures just slightly above 0 °C prepares the plant for sub-zero conditions [47]. In rice farming, varieties grown in temperate regions of the world are particularly sensitive to cold stress as it causes pollen sterility and large reductions in grain yield.

#### 2.1.2. Nutritional Stress in Plants

Nutrients are obtained from the soil through plant roots, but there are many factors that might affect nutrient acquisition, including soil texture and moisture, pH, temperature and organic matter. Both nutrient limitation (or low bioavailability) and nutrient excess might cause stress to the plant and negatively affect physiological and metabolic processes essential for plant growth, development, reproduction and yield. In this scenario, plants must actively respond to maintain nutrient levels within the optimal range. Plants adapt to nutrient stress, i.e., a deficiency or excess of nutrients, by altering numerous physiological, biochemical, and molecular processes [48].

Plant nutrients include macronutrients, which are required in relatively large amounts, and micronutrients, which are required in much smaller amounts. The macronutrients that plants need include nitrogen (N), phosphorus (P), and potassium (K). N serves as a fundamental constituent in key molecules, like amino acids, nucleic acids, and proteins. The efficiency of N acquisition (or nitrogen use efficiency, NUE) is determined by the activity of N transporters involved in N uptake and by the root architecture [49,50]. Regarding P, it is a component in key molecules, such as nucleic acids, phospholipids, and adenosine triphosphate (ATP). It also plays vital roles in enzymatic reactions and signal transduction cascades, particularly protein phosphorylation. P is absorbed through the roots in the form of inorganic phosphate (Pi), and although the overall content of P in the soil is generally high, its low bioavailability represents a limiting factor for plant growth in most agricultural soils. Furthermore, although considerable progress has been made in our understanding of the mechanisms underlying the adaptation of plants to Pi limiting conditions during the past few years, less is known about adaptive mechanisms to Pi excess conditions. As for K, it is involved in different cellular processes controlling photosynthesis and chloroplast function, stomata-opening control, and turgor pressure regulation [51]. This element functions as a cofactor in enzymatic reactions. Despite the abundance of K in most soils, only a small portion can be readily absorbed by plants. Detection of K deficiency triggers the activation of K channels and transporters in the plant, which is sometimes accompanied by the induction of other cellular processes that are also regulated by Ca^2+^, hormones, and ROS [52].

Sulfur (S), magnesium (Mg), and calcium (Ca) are also essential plant nutrients, although they are required in smaller quantities than N, P, and K. For instance, S is a component of important biomolecules, such as amino acids, chlorophylls, phytochelatins, and vitamins, among others [53]. As for Mg, this nutrient controls a wide set of biochemical and physiological processes, such as enzyme activation, nutrient homeostasis and transport, photosynthesis, seed germination, and stomata regulation [54]. Deficiencies in Mg might cause a severe reduction in plant growth and yield. Regarding Ca, this nutrient has a dual function, as it is a structural element in cell walls and membranes, and an intracellular second messenger; strict regulation of Ca uptake, distribution, and storage within the plant is essential to effectively fulfill both functions [55].

Micronutrients also play an important role in balanced crop nutrition. They include iron (Fe), copper (Cu), manganese (Mn), zinc (Zn), boron (B), chlorine (Cl), molybdenum (Mo), and nickel (Ni). Many of these elements function as cofactors in enzymatic reactions. Critical plant functions can be limited by deficiencies in micronutrients, leading to severe reductions in plant growth and yield. Fe is involved in redox homeostasis and photosynthesis, as well as in plant immunity [56]. Cu regulates germination, growth, and photosynthesis, and is essential for the function of ROS-related enzymes [57]. Mn participates in processes associated with photosynthesis, hormone signaling, respiration, and pathogen defense [58]. Zn has a vital role in carbohydrate metabolism, gene regulation, hormone regulation, membrane stability, photosynthesis, and protein biosynthesis [59]. B is important in carbohydrate and protein biosynthesis, cell division and elongation regulation, membrane integrity, nitrogen fixation, and reproductive growth, as well as in responses to abiotic and biotic stressors [60]. Cl participates in cell turgor, volume, and expansion, photosynthesis, and water use efficiency (WUE) and NUE [61]. Regarding Mo, this element functions in hormone biosynthesis, nitrate assimilation, purine degradation, and sulfite detoxification [62]. Ni plays a role in nitrogen metabolism, seed germination, photosynthesis, and vegetative and reproductive growth [63]. Lastly, although selenium (Se) is not believed to be indispensable for plants, this element is known to function as an antioxidant, thereby enhancing tolerance to abiotic stresses, such as salinity, drought, extreme temperature, and toxic metals [64,65]. Se has also an effect on the uptake, transport, and/or accumulation of essential micronutrient elements, such as Fe, Zn, Cu, Mn, and Ni [66].

Calcium (Ca) is also an essential nutrient that plays a dual role in plants. It is required for structural functions in the cell wall and membranes and as a secondary messenger in signal transduction pathways involved in several physiological processes [67]. Ca levels increase in response to different stimuli, i.e., the so-called Ca-signature for the activation of downstream events, including the activation of protein kinases. This, in turn, leads to phenotypic responses related to stress tolerance [68,69,70]. Elevation in calcium concentration in plant cells is an early event upon pathogen infection [71]. Dissecting Ca-mediated signaling cascades is challenging in the context of changing environmental conditions, as intricate positive and negative regulations might occur in response to specific stresses. For instance, high temperatures suppress SA synthesis, thereby compromising immunity [72].

Some other elements, when at low concentrations, are also recognized as beneficial to plants, such as silicon (Si), cobalt (Co), and sodium (Na). In particular, Si is gaining significant recognition in agriculture due to its advantageous effects on plants growth and ability to withstand abiotic stresses [73]. Si is being used as a biofertilizer because it enhances photosynthetic performance and plant growth. In combination with AMF, Si can help alleviate some environmental stressors, such as drought and salinity [74,75]. Co is a cofactor of certain enzymes, thus contributing to metabolic functions in plants, and a component of vitamin B12 (cobalamin), which is required in processes related to nitrogen fixation [76]. On the other hand, although Na does not serve a vital function in the growth, development, or reproductive activities of various terrestrial plants, it can be beneficial under conditions of low K concentration [77]. Finally, although aluminum is commonly seen as a major threat to plant growth, research indicates that aluminum may have beneficial effects in some plant species, including the stimulation of plant growth and mitigation of abiotic and biotic stress [78].

Metals with relatively high densities are regarded as heavy metals. Contamination of agricultural soils with heavy metals is associated with the rapid development of urbanization, industrialization, manufacturing, and mining, which pose risks to human health [79]. Heavy metals that commonly contaminate agricultural soils include Ni, Cd, Hg, Cu, Cr, As, Pb, and Zn [80]. Some heavy metals are considered essential nutrients (Fe, Co, Zn) due to their metabolic functions. However, when in excess, heavy metals trigger oxidative stress and nutrient imbalances which might be detrimental to the plant [81]. Thus, excessive amounts of heavy metals in the soil might reduce the uptake and translocation of nutrients by the plant [82]. Heavy metals in soil reduce seed germination and cause stunted growth, leaf chlorosis, and root browning [81].

Globally, plants are facing large changes in nutrient availability. Not only climate change but also anthropogenic activity are currently driving important alterations in soil nutrients to which plants must adapt [83]. The introduction of chemical fertilizers in agriculture was a major component of the Green Revolution initiated in the 1960s. Nowadays, agricultural ecosystems are often supplemented with external nutrient sources, like chemical fertilizers. For instance, fertilizers containing a mixture of NPK are routinely used in modern agriculture to optimize crop yield. On the other hand, the deficiency of micronutrients is increasingly becoming an important limiting factor in intensive crop production systems [84]. The overuse of fertilizers has not only led to a scenario of nutrient excess in agricultural soils but also raises serious concerns about adverse environmental impacts [48].

#### 2.1.3. Flooding Stress and Light Stress

Excessive water in soil due to heavy precipitation or poor drainage might cause flooding or waterlogging, leading to hypoxia, anoxia, impaired gaseous exchange, and increased ROS production and oxidative damage. Most land plants can be seriously affected by the total or partial submersion of aerial parts, blocking respiration and photosynthesis. Molecular and physiological processes that enable plants to thrive in waterlogged conditions have been recently reviewed [85]. Climate change is also affecting the frequency of heavier rainfall, which is more erratic and unpredictable, thus increasing flood risks in agricultural production [86].

Lastly, light intensity and quality might also be a source of stress for plants. Insufficient light provokes reduced photosynthesis rates, whereas excess light intensity results in photodamage. Therefore, plants have developed protection mechanisms to cope with light stress [87]. Ultraviolet (UV) radiation promotes an increase in ROS levels that cause damage to cellular structures and biomolecules (e.g., peroxidation of lipid membranes, protein and DNA damage) [88].

### 2.2. Biotic Stress in Plants

In nature, biotic stress occurs as a result of the invasion of plants by other living organisms, including pathogenic microorganisms (bacteria, fungi, viruses) along with herbivores, insects, nematodes, and weeds. Diseases caused by biotic stressors result in important reductions in crop yield and, hence, in vast economic losses. To adapt to these diverse biotic stresses, plants have evolved sophisticated mechanisms to detect pathogenic organisms and activate the appropriate response depending on their characteristics (discussed in Section 3.1). Given its socioeconomic relevance, in this review we focus on biotic stresses in rice, with an emphasis on diseases caused by microbial pathogens. Moreover, rice has become a model for research on monocotyledonous species, which also means that rice is considered to be a suitable model to study responses to pathogen infection in monocotyledonous plants.

The most important bacterial pathogen affecting rice is *Xanthomonas oryzae* pv. *oryzae* (*Xoo*), which causes bacterial blight disease [89]. *Xoo* penetrates the leaf through wounds or hydathodes and, once it reaches the xylem vessels, it spreads into the plant, causing necrosis from the leaf tip along the leaf veins. Considering fungal diseases, *Magnaporthe oryzae*, *Rhizoctonia solani*, *Helminthosporium oryzae*, and *Fusarium fujikuroi* are the most predominant fungal pathogens affecting rice production. The extent of yield losses can vary based on environmental factors leading to fluctuations across different seasons and regions. In particular, blast disease caused by *M. oryzae* (synonym, *Pyricularia oryzae*) is one of the most devastating fungal diseases affecting global rice cultivation [90,91,92]. *M. oryzae* can infect different tissues in the rice plant, namely leaves, sheaths, nodes, and panicles. This is a hemibiotrophic fungus characterized by an initial phase of growth within the host cells, followed by a necrotrophic phase. At the biotrophic phase, the fungus obtains nutrients from the host cell, while during the necrotrophic stage, the fungus obtains nutrients from killing the host cells.

As a soil-borne pathogenic fungus, *R. solani* infects leaf sheaths, leaf blades, panicles, and tillers, and causes sheath blight disease in rice [93]. On the other hand, brown spot caused by the fungus *H. oryzae* affects the coleoptile, leaves, leaf sheaths, panicle branches, and glumes, resulting in poor grain filling [94]. Bakanae disease (also known as the “foolish seedling disease”) is caused by one or more *Fusarium* species, mainly *Fusarium fujikuroi* (a member of the *Gibberella fujikuroi* species complex) and represents an emergent threat to rice cultivation in many rice-growing areas [95]. Typical symptoms of bakanae include abnormal elongation due to the production of fungal gibberellins and etiolation, crown and root rot, sterility, and the formation of empty panicles. *Fusarium* species can also produce mycotoxins which might be present in rice seeds, thus raising significant food safety issues for human and animal consumption.

Regarding viral diseases, rice black-streaked dwarf virus (RBSDV), rice yellow mottle virus (RYMV), and rice stripe virus (RSV) are the most viral serious diseases, as they can cause great yield losses up to 100% in Asian, African, and American countries, respectively [96]. Regarding insect diseases, stem borers, such as *Chilo suppressalis* (striped stem borer) and *Scirpophaga incertulas* (yellow stem borer), and planthoppers, such as *Nilaparvata lugens* (brown planthopper) and *Sogatella furcifera* (white backed planthopper), are probably the most serious insect pest of rice.

Clearly, different and complementary strategies need to be implemented for the control of rice diseases in an environmentally friendly way by taking advantage of all the resources and technologies currently available. They include the development of resistant cultivars, the application of biotechnological approaches, the exploitation of beneficial microorganisms, and the use of appropriate management practices.

### 2.3. Effects of Simultaneous Abiotic and Biotic Stresses

In nature, plants are confronted with combinations of multiple abiotic and biotic stressors that might occur simultaneously or sequentially. However, most studies conducted to date on environmental stress in plants focused on plants exposed to individual stressors, while less research effort has been dedicated to exploring combinations of multiple stresses in plants [4,5]. Furthermore, although the molecular mechanisms involved in plant defense responses to individual abiotic or biotic stress factors have been elucidated in great depth, how signaling pathways induced by abiotic stress converge to biotic stress responses, and, vice versa, how biotic stress influences abiotic stress responses, still remain obscure. The responses of plants to combined stress cannot be directly predicted from simply studying one stress at a time, a situation that is rarely found in nature. Increasing evidence supports that signaling pathways that are activated by a particular stress might interact with the plant response to other types of stress in a synergistic or antagonistic manner [6,97].

Simultaneous abiotic and biotic stress might affect physiological, morphological, and molecular processes according to the severity and duration of each type of stress. Such interaction between environmental stresses involves crosstalk between their respective signaling pathways. Stress tolerance mechanisms under combined abiotic and biotic stresses include common/shared and unique responses. In certain cases, however, exposure of plants to a mild stress condition enhances the acclimation to a subsequent exposure to the stress through the acquisition of memory [98]. This phenomenon, known as priming, might enhance the plant’s tolerance to future stress in the same generation and/or in the next generation [99]. The preservation of a primed state over time forms the basis of stress memory, where the capability of a plant to store stress information upon exposure to a stressor prepares the plant for possible future stress exposure. This capability enables the plant to better adapt to shifting environmental conditions [100]. It is also true that the impact of a particular stress on the plant response to subsequent stresses cannot be predicted. For instance, the combination of salt stress and heat stress has a higher impact on growth, chlorophyll content, and Na^+^/K^+^ balance in Arabidopsis plants than the application of these stresses separately [101].

Abiotic stresses, particularly drought and heat, might interfere with host defense responses to pathogen infection. In the model plant *A. thaliana*, elevated temperatures have been shown to hinder defense responses to *Pseudomonas syringae*, but they were enhanced under water shortage [102]. Other abiotic stressors, such as high light, heat, cold, and drought, might also provoke different effects on plant defense against bacterial (*P. syringae*) and fungal (*Botrytis cinerea*) pathogens [103] in Arabidopsis plants. In other studies, it was reported that exposure to temporary heat stress decreases the resistance of *A. thaliana* to *P. syringae* [104], whereas cold exposure decreases susceptibility to this pathogen [105]. As an additional complexity, the expression of immune responses after sequences of abiotic–biotic stress appears to be dependent on the lag phase between abiotic stressors and pathogen infection in Arabidopsis plants [103].

As drought stress impairs plant cell functioning, it might reduce plant defense capabilities against pathogens, thereby affecting disease resistance [106]. In this respect, transcriptome data support the existence of crosstalk between signaling networks under drought and pathogen stress. Also, the intensity and temporality of drought might determine the outcome of plant–pathogen interactions. In Arabidopsis, moderate drought stress was found to enhance susceptibility to *P. syringae* [107], whereas, in common bean, drought exacerbates the root rot caused by *Fusarium solani* [108]. An interesting example of such crosstalk is that, in rice, drought stress enhances susceptibility to infection by the blast fungus *M. oryzae* and the bacterial pathogen *X. oryzae* pv *oryzae* (*Xoo*) [109,110]. An increase in temperature was reported to enhance susceptibility to infection by *F. fujikuroi*, the causal agent of bakanae disease in rice [111]. Because drought episodes are expected to become more frequent with climate change, research efforts to address combined effects of drought and pathogen infection in crop species are of special interest.

Clearly, the outcome of plant–pathogen interactions under abiotic stress would depend on the complex interaction between defense mechanisms and responses to environmental cues, with these responses being also dependent on the intensity and sequence of stress exposure. Because ROS play a key role in the response of plants to abiotic and biotic stresses, maintenance of ROS levels is likely to be important for plant adaptation to biotic and abiotic stress combinations, an aspect that remains poorly explored. Not only genes related to protection against oxidative stress, but also other genes, or general stress-responsive genes, might potentially operate in the plant response to multiple biotic and abiotic stresses. Among them, there are genes related to hormone signaling, genes involved in photosynthesis, senescence-related genes, and *Pathogenesis-Related* (*PR*) genes [112].

### 2.4. Impact of Plant Nutrition in Adaptation to Environmental Stresses

An adequate supply of nutrients is essential for normal growth, development, and reproduction in plants. Soil nutrient availability determines the nutritional status of a plant. Nutrient availability, either deficiency or excess, might also be a source of stress in plants [113]. In particular, proper nutrition can be an important factor in mitigating the effects of abiotic stresses, through the activation of mechanisms involved in detoxification of ROS, structural protection and membrane stability, increased photosynthetic activity, and the activation of stress-associated genes [10]. Plant nutrition can also alleviate abiotic stress through processes that are not directly linked to stress tolerance (i.e., strengthening the root system or increasing leaf expansion or water use efficiency). Along with this, the application of nutrients, either macronutrients or micronutrients, has proven to be effective to alleviate abiotic stress in plants [10].

On the other hand, a shortage of any mineral nutrient might restrict shoot and root growth and provoke early defoliation. However, when the supply of a particular nutrient is increased (e.g., by overfertilization), this increase might cause toxicity to the plant, which will result in a decline in yield. Therefore, to achieve the optimal growth and potential yield of crops, not only nutrient deficiencies but also the excess of nutrients need to be taken into consideration. Due to nutrient–nutrient interactions, deficiency or excess of a particular nutrient might well affect the status of other nutrients in the plant. The availability of a particular nutrient may also have opposite effects on different diseases depending on the host plant and the pathogen: it can increase disease incidence in a particular plant species while decreasing disease incidence in other species [8].

In agriculture, fertilization practices have long been recognized to be important for plant disease management. Although considerable progress has been made during the past years in our understanding of the regulatory mechanisms underlying adaptive processes to nutrient deficiencies in plants, less is known about adaptive mechanisms to nutrient excess conditions. Furthermore, the impact of nutrient supply on disease resistance appears to vary depending on the identity of the interacting partners (host and pathogen), and the type of nutrient and/or nutrient imbalances. This issue is further discussed in Section 4.

### 2.5. Interactions with Beneficial Microorganisms for Improving Multiple Stress Tolerance in Plants

The interaction of plants with beneficial microorganisms, such as plant growth-promoting rhizobacteria (PGPB) and arbuscular mycorrhizal fungi, can improve tolerance to abiotic stress and/or resistance to pathogen infection in plants [114,115,116]. In symbiosis, microorganisms support plants by helping in nutrient absorption, and in turn, the host plant supports the growth of the microbial partners by providing carbohydrates produced through photosynthesis. Positive effects of beneficial microbes on plants relate to their ability to improve plant nutrition and defense priming against a wide range of environmental challenges [117]. Indeed, plant symbiotic interactions with rhizosphere microorganisms are one of the important adaptation strategies used by plants to enhance nutrient acquisition under nutrient-limiting conditions. As an example, mycorrhiza-induced disease resistance is associated with priming of plant defense responses for enhanced resistance to below-ground and above-ground pathogens. A protective effect of root colonization by arbuscular mycorrhizal fungus against infection by the rice blast fungus *M. oryzae* has been described [118]. The arbuscular mycorrhizal symbiosis also enhances tolerance to abiotic stresses, such as drought and salt stress, in rice [119,120]. For an overview of the benefits that plants can receive from their interaction with beneficial microorganisms (e.g., improvement of pathogen resistance and tolerance to abiotic stressors), we refer to Ali et al. (2023) [116]. At present, arbuscular mycorrhizal fungi and PGPB are being increasingly used as biostimulants, which offers a valuable alternative to improve plant nutrition and stress tolerance in crop species, thus allowing an increase in crop yields in a sustainable manner [121].

## 3. Mechanisms Regulating Immunity in Plants

### 3.1. The Plant Immune System

In nature, plants might encounter a large number of microorganisms, but only a few of them are pathogenic to plants. Plants have developed intricate mechanisms to defend themselves against pathogens, which include passive and active defense mechanisms. Passive defense mechanisms are those that are present before contact with the pathogen, consisting of pre-formed structural/physical barriers and the accumulation of certain antimicrobial compounds, whereas active defense mechanisms are activated upon pathogen recognition and constitute the so-called innate immune system [122,123].

As for passive defense mechanisms, the plant cell wall represents the first line of defense against pathogens, acting as a physical and defensive barrier. Upon infection, the plant cell wall usually undergoes dynamic remodeling as a mechanism to prevent pathogen infection [124]. Typical responses to infection by fungal and bacterial pathogens include the reinforcement of cell walls through lignin accumulation and callose deposition. In leaves, epidermal cells are covered by a waxy cuticle whose hydrophobic properties avoid the accumulation of standing water on the leaf surface that can prevent the germination of spores from fungal pathogens. If passive defenses are breached, plants activate innate immune responses [123].

The plant innate immune system efficiently detects potential microbial pathogens, which results in massive transcriptional reprogramming of gene expression [125]. Depending on the molecules that are recognized by the host plant, two immune systems have been defined. Recognition of conserved molecular signatures from invading pathogens, known as pathogen-associated molecular patterns (PAMPs) by host-encoded surface receptors (pattern recognition receptors, PRRs) triggers the activation of a general defense response referred to as pattern-triggered immunity (PTI) [126,127]. Plants also recognize endogenous host-derived elicitor molecules that are released upon infection, generally composed of the cell wall or extracellular protein fragments, peptides, nucleotides, and amino acids. Recognition of these molecules, known as damage-associated molecular patterns (DAMPs) also activates PTI responses [128]. PTI acts against a diverse array of pathogenic microorganisms, such as bacteria and fungi. Early responses during PTI include the generation of reactive oxygen species (ROS), an influx of Ca^2+^ into the cytoplasm, and the activation of phosphorylation signaling cascades, in which wall-associated kinases (WAKs), mitogen-activated protein kinases (MAPKs), and calcium-dependent protein kinases (CDPKs) operate, ultimately triggering the induction of defense-related responses [127]. As a consequence of host−pathogen co-evolution, however, pathogens have evolved to counteract PTI by delivering virulence factors, also known as effectors, into the plant tissue. These effector proteins either prevent pathogen recognition or suppress the host’s defense response, leading to susceptibility.

Plants developed another layer of defense system against microbial effectors in the form of intracellular receptors encoded by resistance (*R*) genes. These receptors are capable of detecting the presence of pathogen effectors, thereby activating effector-triggered immunity (ETI) [126]. ETI is associated with prolonged and robust immune reactions, resulting in finetuned localized host cell death and hypersensitive response (HR), a form of programmed cell death that prevents the spread of infection.

One of the earliest responses of plants to pathogen recognition is the production of reactive oxygen species (ROS), which regulate plant immunity [129]. ROS include hydrogen peroxide (H_2_O_2_), hydrogen radicals (·OH), superoxide anions (O^2−^), and singlet oxygen (^1^O_2_). Among ROS, H_2_O_2_ has multiple functions in plant defense, acting as an antimicrobial agent against invading pathogens and promoting the crosslinking of cell wall components. ROS also act as signaling molecules to activate defense mechanisms.

Nitric oxide (NO) is also recognized as a multifaceted signaling molecule in plant responses to pathogen infection [130,131,132]. Here, recognition of PAMPs and DAMPs triggers the production of NO, leading to the activation of immune responses and local and systemic signaling. Indeed, the synergistic interaction of ROS and nitric oxide (NO) is essential to initiate cell death mechanisms in plants. Additionally, NO can mediate root growth and development at different N supply levels [132,133,134]. NO can also promote the interaction of plant roots with beneficial microorganisms (e.g., rhizobia and arbuscular mycorrhizal fungi) to improve P and N nutrition [132].

However, an excess of ROS can be detrimental to the plant cell by provoking the oxidation of biomolecules, such as proteins, nucleic acids, and membrane lipids, with the latter resulting in membrane damage and, eventually, cell death [135]. To ensure adequate ROS levels, plant cells have scavenging systems to prevent ROS from reaching damaging levels during pathogen infection, which include enzymatic (e.g., superoxide dismutases, peroxidases, catalases, ascorbate peroxidases, and glutathione peroxidases) and non-enzymatic (e.g., ascorbate, glutathione, flavonoids) systems [136]. 

Pathogen-induced signaling cascades in PTI and ETI ultimately lead to the production of antimicrobial compounds, such as melatonin, flavonoids, phytoalexins, polyamines, phenolics, and the induction of defense-related genes, including pathogenesis-related (*PR*) genes [30,137]. PR proteins comprise proteins that exhibit antimicrobial activity, such as chitinases, β-1,3-glucanases, defensins, or thionins [137]. Furthermore, plants produce short endogenous elicitor peptides (PEPs) in response to pathogen attack, which might have multiple functions in activating the innate immune response [138]. However, our current understanding of the role of plant PEPs in immune responses is still limited. Although PTI and ETI are generally classified as two defense layers, crosstalk between these two immune pathways is known to occur. In this way, immune pathways triggered by pathogen recognition, either by cell surface or intracellular receptors, might synergistically enhance each other’s effects, resulting in robust defense mechanisms against pathogens [139,140].

The zig-zag model originally described by Jones and Dangl [126] presents the successive steps of the interaction between the plant and its pathogen. According to this model, pathogens are continuously generating new effector proteins to suppress ETI, while plants evolve novel *R* genes towards these effectors. Although widely used, the zig-zag model has limitations. For instance, the model is based only upon interactions recognition between host receptors and microbial effectors, and recognition of endogenous elicitors, such as DAMPs, is not accounted for in the original zig-zag model [141].

As part of the defense mechanisms that plants develop against pathogen infection, they can develop systemic resistance, defined as systemic acquired resistance (SAR) and induced systemic resistance (ISR). SAR is induced upon pathogen recognition and protects the plant against a broad spectrum of pathogens following an initial infection [142]. Here, the memory of a previous infection can prepare or “prime” the plant for resistance to subsequent pathogen attacks. Unlike SAR, which is induced by direct pathogen infection, ISR is triggered by certain beneficial microbes (i.e., non-pathogenic rhizobacteria, arbuscular mycorrhizal fungi) or certain chemical compounds [143,144].

Plant hormones, which are essential for plant growth and development, also play a central role in orchestrating plant immune responses. The plant hormones salicylic acid (SA), jasmonic acid (JA, and JA-derivatives), ethylene (ET), and abscisic acid (ABA) have long been recognized to play a central role in plant immunity [145]. Auxin and brassinosteroids, and, more recently, strigolactones, have also been described to play a role in plant responses to pathogen infection [146]. Crosstalk among different classes of hormones may enhance resistance against a particular pathogen but could also have a negative effect on resistance. In this way, antagonistic or synergistic interactions among diverse hormone signal transduction pathways enable a plant to finely regulate its immune response to any invader encountered [147]. The overall effect of hormone regulation in disease resistance depends on the lifestyle and infection biology of the invading pathogen (e.g., necrotroph vs. biotroph) and/or specialized mechanisms of each interaction, such as host defense mechanisms and the pathogenicity program in the pathogen [145]. As an example, ET, JA, and SA contribute to blast resistance in rice, whereas ABA contributes to susceptibility [148,149,150,151].

### 3.2. Role of microRNAs in Plant Immunity

Historically, the plant defense response to infection by fungal and bacterial pathogens has been considered to rely on the function of protein-coding genes, while antiviral defense mainly relies on RNA-based mechanisms. Nowadays, it is generally accepted that small, non-coding RNAs also play a crucial role in the regulation of gene expression in processes contributing to resistance against fungal and bacterial pathogens [152,153,154].

In plants, small RNAs can be broadly classified in two major classes: microRNAs (miRNAs) and small interfering RNAs (siRNAs). They can be distinguished based on their function, mechanisms of biogenesis, and mode of action [155]. Whereas miRNAs derive from single-stranded RNAs forming an imperfectly base-paired hairpin–loop structure, siRNAs are generated from perfectly complementary long double-stranded RNAs (dsRNAs).

**Figure 2:**
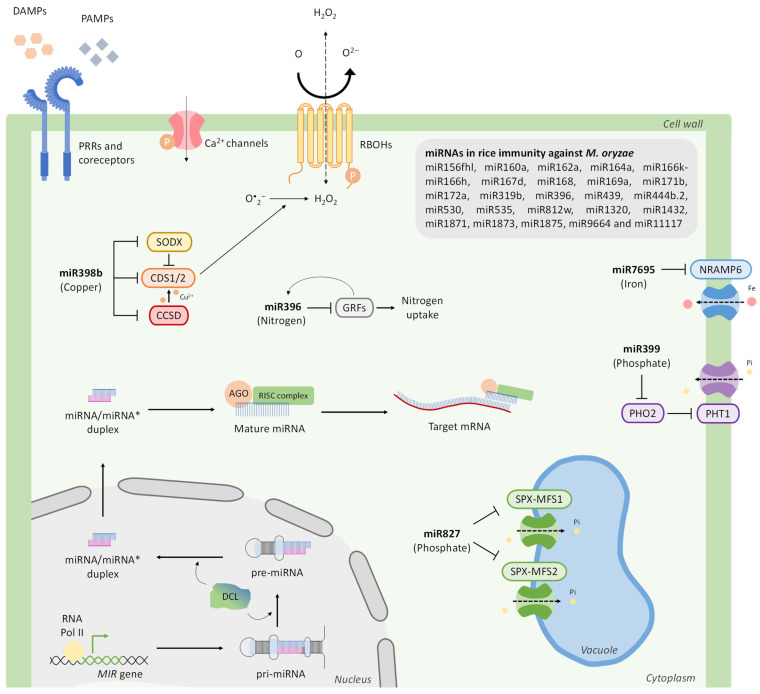
MiRNAs involved in the regulation of nutrient homeostasis and immunity against the rice blast fungus *M. oryzae* are shown. A schematic representation of canonical miRNA biogenesis pathway is shown. MiRNA biogenesis is a multistep process in which primary precursor transcripts (pri-miRNAs) are cleaved into pre-miRNA precursors and miRNA duplexes (miRNA/miRNA*) by the activity of DICER-LIKE1 (DCL) proteins. MiRNAs are exported from the nucleus to the cytosol where the functional strand of the miRNA/miRNA* duplex exerts its regulatory function on target transcripts, either transcriptional regulation or translational repression. The miRNA target genes and the biological processes that they regulate are indicated. AGO, ARGONAUTE; CCSD, copper chaperone for superoxide dismutase; CDS, Cu/Zn-superoxidase dismutase; DAMPs, damage-associated molecular patterns; GRFs, growth-regulating factors; NRAMP6, natural resistance-associated macrophage protein 6; PAMPs; pathogen-associated molecular patterns; PHO2, phosphate 2; PHT1, phosphate transporter 1 family; pre-miRNA, precursor microRNA; PRRs, pattern recognition receptors; RBOHs, respiratory burst oxidase homologs; RNA Pol II, RNA polymerase II; SODX, superoxide dismutase X; SPX-MFS, SYG1/PHO81/XPR1-major facilitator superfamily.

MicroRNAs (miRNAs) are 20–24 nucleotides in length and direct gene silencing at the post-transcriptional level through sequence-specific cleavage or translational repression of target mRNAs [156,157]. They are transcribed from *MIR* genes as long precursor transcripts or primary miRNA (pri-miRNAs) that adopt a stem–loop structure that is processed by RNAse III Dice-like (DCLs) enzymes, mainly DCL1 enzymes, and give rise to a miRNA/miRNA* duplex (miRNA-5p/miRNA-3p) [158] (Figure 2). In most cases, one strand of the miRNA duplex is functional, while the other is degraded. The functional strand of the duplex is then loaded into an ARGONAUTE (AGO)-containing RNA-induced silencing complex (RISC) that guides miRNAs to target mRNAs to regulate gene expression.

MiRNAs play a significant role in diverse developmental processes as well as in adaptive responses to abiotic and biotic stresses. Distinct miRNAs have been shown to play a central role in regulating the plant’s immune response, both PTI and ETI [159,160,161,162]. Although a large number of miRNAs have been reported to be responsive to pathogen infection in different plant species, the exact mechanism by which most of these pathogen-regulated miRNAs modulate plant immune responses has not been elucidated. Defense-related miRNAs have been described and functionally validated in the model *Arabidopsis thaliana* as well as in crop species, including rice [163,164,165,166,167,168,169]. These miRNAs might function as positive or negative regulators of plant immune responses depending on the function of their target gene(s). Some miRNAs are regulators of hormone biosynthesis and/or signaling pathways regulating plant defense responses, such as SA, ET, and JA. MiRNAs whose accumulation is regulated in different plant–pathogen interactions have been reviewed [154,170,171]. As miRNAs function as fine-tuners of target gene expression, they can directly or indirectly regulate processes involved in multiple environmental stress responses.

The rice/*M. oryzae* system has received much attention in the characterization of miRNAs regulating disease resistance and/or susceptibility, likely because of the significance of blast caused by this fungus in rice. Along with this, the accumulation of a large number of miRNAs has been found to be altered during *M. oryzae* infection or treatment with elicitors prepared from this fungus [164,172,173]. Rice miRNAs with a known function in blast resistance/susceptibility are shown in Figure 2. They include: miR156fhl, miR160a, miR162a, miR164a, miR167d, miR168, miR169a, miR171b, miR172a, miR319b, miR396, miR398, miR399, miR439, miR444b.2, miR530, miR535, miR812w, miR827, miR1320, miR1432, miR1871, miR1873, miR1875, miR7695, miR9664, miR11117, and the polycistronic miRNA miR166k-166h [165,174,175,176]. An additional complexity in studies on miRNAs in plant–microbe interactions is that the regulation and function of a particular miRNA might differ among plant species, also depending on the type of pathogen. For instance, *MIR398* overexpression in rice enhances resistance to *M. oryzae*, whereas its overexpression in Arabidopsis compromises resistance against the bacterial pathogen *P. syringae* [177,178].

### 3.3. Modulation of Plant Immune Responses by Pathogens

In addition to the overall plant defense response to pathogen attack, pathogens have also evolved ingenious mechanisms to overcome the plant immune response. As previously mentioned, pathogens produce effector proteins as invasion weapons to suppress plant defense responses and propagate in the host tissue. These effectors are produced by fungi, bacteria, and oomycetes, and are either secreted to the plant apoplast or delivered into the plant cell [179]. In addition to suppressing plant immunity, pathogen effectors can interfere with diverse cellular functions in the plant. To increase pathogenicity, some pathogens produce molecules that are functionally or structurally similar to those produced in the host plant, such as hormone-like molecules, and peptides [180,181,182]. For instance, during the biotrophic phase of host tissue colonization, the rice blast fungus *M. oryzae* secretes an analog of JA (12-hydroxy jasmonic acid, 12OH-JA) that disables the JA-mediated defense signaling in the plant [183]. In addition to JA, *M. oryzae* has the ability to produce cytokinin and ABA that also facilitate pathogenesis and host invasion [184,185]. The fungus *F. fujikuroi* (causing bakanae) produces gibberellins that results in abnormal elongation in rice seedlings that provides the pathogen additional space and nutrients. Other microbial molecules that mimic those present in the plant are the rapid alkalinization factors (RALF)-like peptides which, like the plant RALFs, induce alkalinization of the extracellular compartment in plant tissue. Microbial RALFs hijack ligand-receptor interactions, thus interfering with host immunity [180,182,186]. The pathogenic fungus *B. cinerea* secretes an exopolysaccharide that functions as an elicitor of the host SA pathway to eventually suppress the JA-mediated signaling during infection of tomato plants and promotes disease development [187].

On the other hand, as pathogens require nutrients from the host to sustain growth in the infected tissue, it is not surprising that pathogens use effector proteins to manipulate the plant nutrient metabolism for their own benefit. Strategies developed by pathogens to provide themselves with nutrients might promote disease development [188]. For instance, many pathogens need to acquire sugars and other metabolites from the apoplast and, when needed, pathogens produce effector proteins to convert sugars into their preferred types which are then taken up by the pathogen. This, in turn, allows the pathogen to better grow in the apoplast [189]. Pathogens can also increase the production of specific nutrients in plants by delivering effectors that regulate the biosynthesis of such nutrients [188]. Certain pathogens, such as *P. syringae* pv. tomato DC3000, *Cladosporium fulvum*, and *Ralstonia solanacearum*, produce effectors that induce the synthesis of the non-protein amino acid gamma-aminobutyric acid (GABA) which then serves as a nutritional source to the pathogen during host colonization [190]. Together, these observations highlight the wide variety of strategies that phytopathogens use to promote virulence by either attenuating plant immunity or interfering with host cellular processes to facilitate infection.

### 3.4. Cross-Kingdom Small RNA Trafficking Between Plants and Pathogens Modulates Disease Resistance

Reflecting the evolving nature of plant–pathogen interactions, small RNAs (siRNAs and miRNAs) have been shown to exert regulatory functions in a cross-kingdom fashion. Here, plants and phytopathogens have evolved mechanisms to silence genes in the interacting organism. The transfer of small RNAs from a pathogen to the host plant can suppress plant immune responses, with these small RNAs acting as classical protein effectors [153,191,192,193,194]. The first reports of pathogen small RNAs acting as effectors to inhibit host immunity came from studies in *A. thaliana* and tomato during interaction with *B. cinerea*. Here, *B. cinerea* produces small RNAs which move into the plant cell to selectively suppress host defense responses [191,192]. Since then, pathogen-derived small RNAs capable of regulating host immunity via trans-kingdom RNA silencing have been described in diverse plant-pathogen interactions, mainly plant–fungus and plant–oomycete interactions [195,196,197,198,199]. Cross-kingdom movement of small RNAs between plants and pathogens is bidirectional [195,200]. Whereas small RNAs from pathogens move into plant cells to suppress host immunity, plant small RNAs can travel to pathogens to inhibit their virulence. The exchange of small regulatory RNAs between plants and pathogens takes place through extracellular vesicles that deliver small RNAs to the interacting partner where they exert their regulatory role in gene silencing [201,202]. Both siRNAs and miRNAs can mediate cross-kingdom regulation of gene expression to interfere with relevant partner’s functions [203]. Examples of the transfer of small RNAs from plants to pathogens, siRNAs or miRNAs are found in cotton/*Verticillium dahliae*, wheat/*Zymoseptoria tritici*, and wheat/*Puccinia striiformis* f. sp. *tritici* interactions [196,204,205].

## 4. Impact of Plant Nutrition on Disease Resistance

Soil is a major source of nutrients needed by plants for growth. However, nutrient availability in soils might vary greatly according to environmental conditions. To maintain nutrient homeostasis, plants have developed adaptive mechanisms to cope with soil nutrient fluctuations. Emerging evidence suggests that nutrient availability is also crucial for mounting an effective defense against pathogens and that crosstalk between nutrient signaling pathways and immune responses occurs in plants [9,206]. Even though the regulation of nutrient homeostasis and disease resistance has been historically studied separately from each other, evidence gathered during the past years indicated that the plant nutritional status modulates the expression of plant immune responses [8,9]. Adequate and balanced nutrient levels in plants are essential not only to sustain plant growth but also to build an effective immune response in the plant.

During pathogen infection, host and pathogen compete with each other for essential nutritional resources, hence, nutritional stress might affect the outcome of the interaction. Foliar pathogens must acquire nutrients from the host and, accordingly, the availability of nutrients in the host plant can be an important factor contributing to the establishment of the disease. Different results are, however, found in the literature regarding the impact of nutrient stress on disease resistance, which appears to be dependent on the identity of the interacting partners, namely the host and pathogen [11]. A priori, an increase in nutrient supply might create a more favorable environment for pathogen growth to promote pathogenicity. On the contrary, the accumulation of certain nutrients (e.g., iron) can be toxic for the pathogen, thus protecting the plant from infection [20,207]. Accordingly, nutrient homeostasis must be carefully regulated during pathogen infection to allow normal plant growth while providing a way to arrest pathogen growth. Although adaptation to nutrient stress and immunity are not independent processes, the molecular mechanisms involved in nutrient responses and innate immunity remain poorly understood. Additionally, interconnections between nutrients (e.g., the level of a particular nutrient might affect other’s nutrient content) might determine the effect of a particular nutrient on disease resistance. As an example, the antagonistic interaction between Fe and Pi has been noted in the area of plant nutrition [208,209,210]. Interconnections between Pi and Fe signaling might then account for different responses to pathogen infection when their concentrations in the environment vary (such as due to fertilizer application). Studies in rice revealed that Pi accumulation enhances susceptibility to *M. oryzae* infection [12]. In contrast, local accumulation of Fe at the sites of infection enhances blast resistance [20]. Then, reciprocal controls between nutrients and interconnected regulations used by plants to adapt to their environment might determine the outcome of plant–pathogen interactions.

Phytopathogenic fungi have diverse lifestyles and use different strategies to obtain nutrients from their host plants [211]. The impact of nutrient stress on disease resistance might then vary depending on the lifestyle of the pathogen. Broadly speaking, plant pathogens fall into different categories based on their lifestyles, i.e., biotrophs, necrotrophs, and hemibiotrophs. Necrotrophic pathogens kill the host cells and then feed on those dead cells, whereas biotrophic pathogens feed on living cells and cause limited injury. Hemibiotrophs (i.e., *M. oryzae*) have an initial biotrophic phase that is followed by a necrotrophic phase. Because of their different lifestyles, the effectiveness of plant defense in conferring resistance or susceptibility might also vary depending on the nutrient acquisition strategies used by the pathogen and the nutrient availability in the plant niche in which the pathogen grows and multiplies [211,212]. Mineral nutrients and water are abundant in the xylem and the root apoplast, whereas sugars and amino acids are predominant in the phloem and the leaf apoplast (i.e., bacterial pathogens preferentially invade the apoplast, but some can inhabit xylem or phloem) [211,213].

As previously mentioned, fertilizers containing essential nutrients are widely employed in modern agriculture to ensure optimal yields. However, the indiscriminate use of fertilizers may have unintended effects on plants by increasing their susceptibility to diseases. Excessive nitrogen fertilization has long been recognized to heighten susceptibility to *M. oryzae* infection, known as nitrogen-induced susceptibility (NIS) [15]. In Arabidopsis, nitrogen nutrition also alters susceptibility to the necrotrophic fungus *Alternaria brassicicola*, a response which depends on the chemical form of the nitrogen source (e.g., NH_4_^+^ or NO_3_^−^ nutrition) [214]. Thus, NO_3_^−^ nutrition was found to increase the susceptibility of *A. thaliana* to *A. brassicicola* compared to NH_4_^+^ nutrition. This NO_3_^−^-dependent susceptibility was proposed to be the consequence of a higher production of nitric oxide at the infection site and a change in amino acid composition [214]. Contrary to what is observed in rice, in cotton, potato, and tomato, a high nitrogen content confers resistance towards *Alternaria* spp., the causal agent of early blight disease [215].

High phosphate (Pi) fertilization has been shown to increase the susceptibility of plants to *M. oryzae* infection [12], whereas overexpression of *OsPHT8*, a high-affinity Pi transporter in rice, aggravates blast and bacterial blight disease severity [216]. However, while increasing the Pi content in rice enhances susceptibility to *M. oryzae* infection, an increase in Pi level in Arabidopsis plants enhances resistance against the fungal pathogens *Plectosphaerella cucumerina* and *Colletotrichum higginsianum* [12,13,21].

The treatment of rice plants with high Fe enhances resistance against *M. oryzae* [217]. Blast resistance in high iron-treated rice plants was found to be accompanied by the local accumulation of Fe at the infection sites and the upregulation of defense-related genes. Ferroptosis, an iron-dependent cell death process, is a key process in the response of rice plants to infection by virulent *M. oryzae* [20,207]. In Fe-starved *Arabidopsis thaliana*, enhanced resistance to bacterial (*Dickeya dadantii*) and fungal (*B. cinerea*) pathogens was described [14,19]. Of interest, Fe availability leads to antagonistic immune phenotypes in Arabidopsis and rice [218]. Whereas rice uses Fe to trigger ferroptosis, an iron- and oxidative stress-dependent regulated cell death that confers disease resistance, Arabidopsis plants prioritize the proper accumulation of secondary metabolites with defensive properties, i.e., glucosinolates and anthocyanins, during infection with the fungal pathogen *Colletotrichum higginsianum* [20,207,218].

A direct correlation between blast disease resistance and K nutrition was previously reported in rice [19]. Here, the *M. oryzae* effector AvrPiz-t suppresses rice immunity by interfering with K signaling components that are important for both K absorption and host resistance. K deficiency in rice increases the plant’s susceptibility to infection by the fungal pathogens *Sclerotium oryzae* and *Sarocladium oryzae* [219,220]. Conversely, higher levels of K or Mg have been found to reduce susceptibility to sheath blight and brown spot disease caused by *Rhizoctonia solani* and *Bipolaris oryzae*, respectively [16,17]. Increased copper levels provide broad-spectrum resistance against viral infections in rice [221]. Also, reduced levels of sulfate in rice results in increased resistance against *Xoo* and *X. oryzae* pv *oryzicola* (*Xoc*) [22].

Calcium plays a critical role in cellular signaling in numerous processes involved in plant growth and disease resistance. Because calcium moves slowly through exchange in the xylem and is dependent upon water flow, disruptions in that flow can lead to Ca deficiencies. Calcium deficiency occurs when the soil calcium level is low or conditions for calcium uptake are not favorable (i.e., calcium deficiency can be a problem in acid soils). Deficiency of this vital nutrient can make plants more susceptible to diseases caused by various pathogens. In particular, banana wilt disease, a devastating condition caused by the soil-borne pathogen *Fusarium oxysporum* f. sp. *Cubense* (*Foc*), has been increasingly linked to Ca deficiency [222,223].

Mg availability may also vary depending on the environmental conditions. However, compared with other nutrients, less information is available on the effect of Mg on disease resistance in plants. It was reported that Mg deficiency increases the severity of peanut leaf spot caused by *Mycosphaerella arachidis* [224], whereas high levels of Mg significantly increased susceptibility to bacterial spot in pepper and tomato caused by *Xanthomonas campestris* pv. *vesicatoria* [225].

Regarding Si, although this element is considered a non-essential element for plant growth, it regulates biological functions involved in plant development and modulates defense responses to fungal and bacterial pathogens (for a recent review, we refer to Verma et al. (2024)) [226]. Notably, its application has been shown to enhance disease resistance in different crop species and pathogen interactions, including wheat/*Septoria nodorum*, barley/*Alternaria* spp., cucumber/*Phytium* spp., maize/*Pythium aphanidermatum*, pea/*Mycosphaerella pinodes*, coffee/*Cercospora coffeicola*, cherry/*Penicillium expansum* and *Monilinia fructicola*, melon/*Pythium aphanidermatum*, potato/*Fusarium sulphureum*, banana/*Mycosphaerella fijiensis*, and strawberry/*Botrytis cinerea* [226]. Equally, Se treatments have been shown to effectively control plant diseases, such as sclerotinia stem root in rape (caused by *Sclerotinia sclerotiorum*), tomato gray mold (caused by *B. cinerea*), wheat fusarium head blight (caused by *Fusarium graminearum*), and black shank in tobacco (caused by *Phytophthora nicotianae*), among others [227,228,229,230].

Collectively, these pieces of evidence support the existence of interconnections between nutrient signaling and immune signaling in plants, while illustrating the relevance of plant nutrition in determining the outcome of plant–pathogen interactions.

The nutrient supply influences not only plant immune responses but also fungal pathogenicity. During nitrogen-induced susceptibility in rice, the fungus adapts to high N conditions by modifying its pathogenicity program to promote blast susceptibility [18]. Fertilization with high Pi was found to be accompanied by stronger expression of the *M. oryzae* effector and pathogenicity mitogen-activated protein kinase (PMK1) genes [23]. Downregulation of *M. oryzae* genes encoding suppressors of plant cell death were down-regulated, whereas plant cell death-inducing effectors were upregulated in rice plants supplied with high Pi [23]. Pi-induced susceptibility to *M. oryzae* infection might then be the consequence of a combination of factors, including the repression of pathogen-inducible immune responses (e.g., ROS accumulation, defense-gene expression) and the potentiation of fungal pathogenicity. The combination of these factors would contribute to higher colonization by *M. oryzae* in rice tissues accumulating Pi [23].

**Figure 3:**
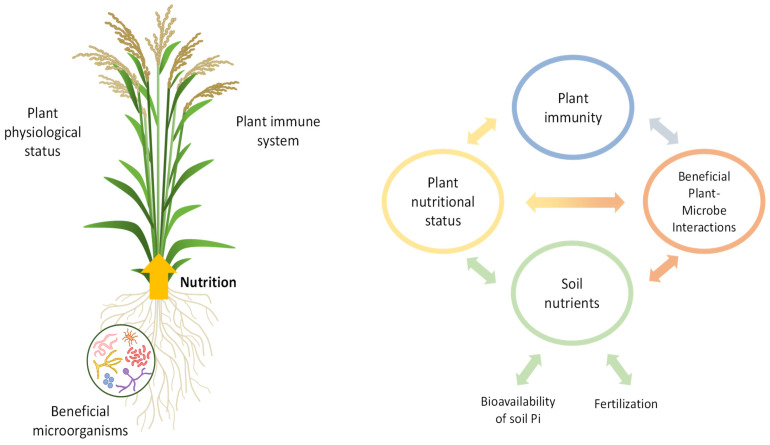
Plant disease resistance depends on many factors, including the type of host–pathogen combination and environmental conditions. Balanced nutrition is an important factor in controlling plant diseases. Nutrient supply might affect disease resistance either by improving plant vigor or by modulating plant defense responses. The bioavailability of nutrients in the soil as well as fertilization practices might also have an effect on the incidence and severity of a particular disease. Beneficial microbes enhance nutrient uptake, improve plant immune responses, and help plants to manage pathogen infection.

Overall, plant disease resistance depends on many factors, including genetic factors and the type of plant–pathogen interaction, as well as environmental conditions (e.g., nutrient availability) and interactions with beneficial microbes (Figure 3). Understanding plant–pathogen interactions requires the use of interdisciplinary approaches, integrating a wide range of disciplines, such as genetics, molecular biology, biochemistry, physiology, phytopathology, microbiology, agronomy, ecology, environmental chemistry, and soil sciences. At present, there is a growing need to develop sustainable agricultural practices to sustain productivity while reducing the use of agrochemicals, fertilizers, and pesticides. This need is further reinforced by legislative and public pressures. To gain a comprehensive understanding of the impact of nutrient stress on disease resistance, it is imperative to conduct further research.

### 4.1. miRNAs Mediate Crosstalk Between Nutrient Signaling and Immune Signaling in Plants

Crosstalk between signaling pathways is essential to integrate the plant response to combined environmental stresses. MiRNAs are excellent candidates for the coordination of plant responses to biotic and abiotic stressors, and certain miRNAs have been shown to exhibit regulatory functions in orchestrating multiple stress responses, including nutrient stress adaptation and pathogen resistance. Furthermore, miRNAs can manifest their silencing activity non-cell-autonomously, as they can exert their regulatory function in tissues distant from those in which they are synthesized. Below, we discuss examples of miRNAs involved in adaptive processes to nutrient stress, as well as miRNAs involved in crosstalk between signaling processes induced by nutrient stress and pathogen infection simultaneously.

MiRNAs can regulate nutrient homeostasis by directly or indirectly governing the expression of transporters involved in nutrient uptake or mobilization. Among them are miRNAs associated with N nutrition (e.g., miR167 and miR393), Pi nutrition (miR399 and miR827), Fe nutrition (miR7695), S nutrition (miR395), and Cu nutrition (miR398, miR397, miR408, and miR857) [231,232]. Moreover, certain miRNAs with a known function in nutrient homeostasis have been demonstrated that might also play a role in the regulation of plant immune responses.

In rice, miR7695 targets and suppresses the expression of *OsNramp6* (*natural resistance-associated macrophage protein 6*) encoding an iron transporter gene and regulates iron homeostasis [164,217] (Figure 2). This particular miRNA also modulates resistance to infection by the rice blast fungus *M. oryzae* [164]. *MIR7695* overexpression, as well as *OsNramp6* silencing, enhances resistance to *M. oryzae* infection in rice plants [164,217,233]. *MIR7695* activation was associated with local iron accumulation at infection sites and a stronger induction of defense-related genes, including *PR* and diterpenoid phytoalexin biosynthesis genes [233]. Equally, exposure of rice plants to high iron enhances resistance to infection by the rice blast fungus *M. oryzae* [20]. Iron and miR7695-mediated regulation of iron distribution facilitate ferroptosis and ROS-dependent defense responses, leading to resistance against *M. oryzae* [20,207,233].

Also in rice, miR395 regulates sulfate assimilation and distribution by targeting ATP sulfurylase *OsAPS1*, *OsSULTR2*;*1*, and *OsSULTR2*;*2* [231]. *MIR395* overexpression reduces the transcript abundance of its target genes and provokes sulphate accumulation in rice leaves. This, in turn, results in the inhibition of bacterial proliferation (*X. oryzae* pv. *oryzae*, *X. oryzae* pv. *oryzicola*) and resistance to bacterial infection [22]. On the contrary, plants with lower miR395 activity showed enhanced bacterial pathogen growth. Taken together, these findings indicate that rice miR395 modulates sulphate metabolism during the interaction of rice plants with bacteria pathogens.

The cross-regulation between Pi signaling pathways and immune signaling in which miRNAs operate is presented for miR399 and miR827 (Figure 2). These particular miRNAs are key components of the phosphate starvation response (PSR) that is activated in response to Pi limitation in plants [234,235]. In rice and Arabidopsis, miR399 targets *PHO2* (*PHOSPHATE2*) encoding a ubiquitin E2 conjugase implicated in the degradation of the plasma membrane transporter PHT1 (PHOSPHATE TRANSPORTER 1) [236]. However, miR827 targets different genes in rice and Arabidopsis. In rice, miR827 targets two vacuolar Pi transporters, namely *OsSPX-MFS1* and *OsSPX-MFS2* [237], whereas in Arabidopsis, miR827 targets *NITROGEN LIMITATION ADAPTATION* (*NLA*) encoding an E3 ubiquitin ligase involved in the regulation of N and Pi homeostasis [238]. PHO2 and NLA mediate the ubiquitination and degradation of the plasma membrane transporter PHT1 in Arabidopsis. Infection by fungal pathogens also causes alterations in miR399 and miR827 accumulation in both rice and Arabidopsis [12,13,21,239]. Pi accumulation caused by *MIR399* overexpression negatively regulates defense gene expression in rice, thus increasing susceptibility to infection by the rice blast fungus *M. oryzae* [12]. Contrary to this, an increase in Pi content caused by *MIR399* overexpression in Arabidopsis positively regulates immune responses which, in turn, increases resistance to fungal infection [13]. Pi accumulating Arabidopsis plants (e.g., wild-type plants grown under high Pi supply and *nla* mutants) showed enhanced callose accumulation and camalexin (the major phytoalexin in Arabidopsis) and accumulation of the defense-related hormone JA under infection conditions [21]. Regarding miR827 (targeting vacuolar Pi transporters), its overexpression enhances susceptibility to infection by *M. oryzae*, which is associated with a weaker induction of defense gene expression during pathogen infection [239]. Conversely, CRISPR/Cas9-induced mutations in the *MIR827* gene completely abolish miR827 production and confer resistance to *M. oryzae* infection [239]. Therefore, not only the regulation of Pi content in leaf tissues but also the cytoplasmic/vacuolar distribution of Pi might be important factors in determining disease resistance in rice plants. Evidence also supports the involvement of other components in the Arabidopsis PSR in modulating the Arabidopsis immune system [240,241].

Collectively, this piece of evidence suggests that certain miRNAs can function as master regulators of signaling pathways induced by nutrient stress and pathogen infection. However, there is still lack of knowledge about the function of miRNAs in the adaptation to combinations of multiple stresses in crop species. For additional information on miRNAs involved in adaptive processes to nutrient stress during plant–microbe interactions, we refer to other published papers [176,232].

### 4.2. Effect of the Plant Nutritional Status on Pathogen Virulence

In plant–pathogen interactions, nutrient supply can affect not only physiological processes in the host plant but also pathogen virulence, and the combination of these factors can shape the dynamics of disease. A priori, balanced plant nutrition would provide more resources for the host to launch effective immune responses. On the other hand, an increase in nutrient supply would increase the availability of nutrients to the pathogen which, in turn, would stimulate pathogen growth in the host tissue. Indeed, the regulation of fungal genes involved in nutrition, mainly carbon and nitrogen, during infection, is well documented [242]. Under this scenario, a balance between the host nutritional status and resources available to the pathogen is expected to determine the fate of their interaction.

A relationship between nutrient supply (e.g., N or Pi supply) and *M. oryzae* pathogenicity has been described in rice. Under high nitrogen fertilization, the expression of *M. oryzae* effector genes and pathogenicity-related genes is increased. The stimulation of the fungal pathogenicity program enhances blast susceptibility [18]. More recently, Pi accumulation in rice tissues was found to dictate the expression of *M. oryzae* Pi transporter genes during infection, suggesting that the fungus is capable of adapting to host Pi levels, presumably to improve fungal nutrition in the infected tissue [23]. In addition to fungal pathogenicity, growing rice under high Pi fertilization fosters the expression of *M. oryzae* effectors and promotes the invasive growth of *M. oryzae* in rice leaves. Furthermore, *M. oryzae* adapts its strategy of infection according to Pi levels in the host tissue by advancing the biotrophy–necrotrophy switch in rice leaves accumulating Pi [23]. In this way, the negative effect that high Pi supply has on blast incidence results from a combination of factors that take place in the two interacting partners. Thus, Pi accumulation decreases the capability of the rice plant to activate defense responses and stimulates the expression of *M. oryzae* effector genes [23]. These observations open the way to further investigate mechanisms involved in nutritional regulation during plant–pathogen interactions. Clearly, a greater understanding of mechanisms underlying plant nutrition, plant immunity, and pathogen virulence is still needed.

## 5. Conclusions and Future Directions

Plant diseases constantly pose a serious threat to crop production and food security. Although substantial evidence links the nutritional status of plants to disease resistance in agricultural environments, the molecular mechanisms underlying these processes have only recently started to be understood. The acquisition of nutrients is crucial for the growth of the two interacting partners and, therefore, nutritional-based mechanisms can shape the interactions between plant and pathogens. During co-evolution, plants evolved defense mechanisms based on sequestration of essential nutrients away from the pathogen, referred to as nutritional immunity. Additionally, plants accumulate certain nutrients that can be toxic to the pathogen, thus protecting the plant from infection. As an example, the localized accumulation of Fe at the sites of infection provokes ferroptosis. On the other hand, pathogens have evolved the ability to deliver effectors into the plant, including those that interfere with host metabolism which, in turn, facilitate nutrient acquisition from the host plant. Whereas much progress has been made on pathogen effectors suppressing host immune mechanisms, our understanding of pathogen effectors capable of modulating the host metabolism is only beginning to emerge. Now an important issue is to understand how nutrient availability influences the deployment of immune responses in the host plant, and how nutrients in the plant might affect pathogen virulence. To achieve this goal, a better knowledge of the interconnected regulations between nutrient signaling and immune signaling in the plant is required.

This review showcases research on miRNAs involved in nutrient homeostasis and disease resistance. As miRNAs function by finetuning gene expression rather than turning gene expression on and off, these regulatory molecules are perfectly suited to act as hubs connecting different signaling pathways, such as those that are activated by nutrient stress and pathogen infection. If so, miRNAs might well function by orchestrating molecular events that enable plants to withstand simultaneous biotic and abiotic stresses in plants. Supporting this possibility, miR399 and miR827 are known to play a crucial role in Pi homeostasis and immune responses. However, many questions remain open, and deciphering miRNA-mediated mechanisms underlying nutrition and disease resistance is an important aspect of future research. Advances in the areas of genome editing technologies (especially the CRISPR/Cas9 system) and synthetic biology will greatly help us to better understand the contribution of miRNAs in nutritional regulation and immunity in crop species for the development of varieties that can tolerate single or multiple environmental challenges. Also, great advances have been made during the past years in the field of cross-kingdom transfer of small RNAs from pathogens to plants and vice versa from plants to pathogens. Small RNA trafficking between the host plants and the attacking pathogen might have significant implication in crop protection. Equally, research on small RNA-based plant protection against diseases will greatly help us to propose new strategies for crop protection.

## 6. Perspectives

Increased food demand impels rice farmers to use agrochemical fertilizers to achieve maximum productivity and high-quality products from the existing arable land area. Although technologies and initiatives adopted during the Green Revolution substantially increased food production, it is also true that the Green Revolution brought the widespread use of fertilizers. This increase in fertilizer use has raised serious concerns about environmental pollution, water eutrophication, and a drastic decrease in soil quality. In particular, the excessive use of fertilizers has led to accumulation of Pi and nitrates in agricultural soils, but so far little attention has been paid to investigating the mechanisms by which plants might adapt to excess of fertilizers. Furthermore, plants often absorb only a small portion of this applied fertilizer, and the unused nutrients in the soil can run off into waterbodies. The effects of the indiscriminate use of fertilizers are persisting, and due to the overuse of fertilizers, newly developed plant varieties usually require far more fertilizer than traditional varieties. Under this scenario, limited information is currently available on the mechanisms that plants might use to counter/offset the effects of excess nutrients. With the predicted global demand and loss of the world’s arable land, continuing indiscriminate usage of agrochemicals is not advisable in an ever-deteriorating environment. Alternative solutions aiming to increase yield with a reduction in the use of agrochemicals need to be implemented.

In rice cultivation, diseases caused by pathogens are responsible for important economic losses. Currently, high rates of pesticides are currently applied in rice cultivation to reduce losses caused by pathogens and, at the same time, fertilizers are widely used to optimize yield. Paradoxically, the overuse of fertilizers (e.g., NPK fertilizer), while contributing to environmental pollution, might have unintended consequences by facilitating blast infection: excessive nitrogen and phosphate fertilization has been shown to increase susceptibility to infection by the blast fungus in rice. This observation also supports cross-talk between signaling pathways induced by N and/or Pi supply and pathogen infection. From all of this, there is still much to know about nutritional regulation in plant–pathogen interactions that might aid in the prevention of diseases in plants. Finding hub genes linked with nutrient stress and immune responses will be one of the hotspots in the research on plant disease resistance in the future. Addressing this question is essential to develop more sustainable approaches to maintain rice production with a reduced input of fertilizers and pesticides.
